# Effects of Naltrexone on Expression of Lipid Metabolism-Related Proteins in Liver Steatosis Induced by Endoplasmic Reticulum Stress in Mice

**DOI:** 10.1155/2022/6572499

**Published:** 2022-05-29

**Authors:** Ping Yang, Leyao Xiao, Fei Zhao, Wei He, Guijuan Zhang, Yongjing Tang, Yinghua Chen, Qijiao Cheng, Yihuai He

**Affiliations:** ^1^Department of Infectious Diseases, Affiliated Hospital of Zunyi Medical University, Zunyi 56300, China; ^2^School of Nursing, Zunyi Medical University, Zunyi 56300, China; ^3^Clinical College of Zunyi Medical University, Zunyi 56300, China

## Abstract

This study aimed to explore the effect of naltrexone on the expression of lipid metabolism-related proteins in liver steatosis induced by endoplasmic reticulum stress in mice. Thirty inbred mice (C57BL/6J) were divided into three groups: group A (normal control group), group B (model control), and group C (naltrexone group). The male mice in group A were fed a regular diet, and the mice in groups B and C were fed a high-fat diet. Liver steatosis was observed by histopathological sections. Mouse liver (alanine aminotransferase (ALT) and triglyceride (TC)) content (glucose regulatory protein (GRP78), endoplasmic reticulum transmembrane protein kinase-1*α* (IRE-1*α*), C/EBP source protein (CHOP), cysteine-containing aspartate proteolytic enzyme 12 (caspase-12), B lymphoma-2 (Bcl-2), and cell death mediator (Bim)) was detected. Compared with group A, bodyweight, fat weight, ALT, TG, and hepatic steatosis were significantly increased in B and C groups (*P* < 0.05); compared with group B, group C showed a significant decrease in bodyweight, fat weight, ALT, TG, and hepatic steatosis (*P* < 0.05). Compared with group A, the expression levels of GRP78, IRE-1*α*, CHOP, caspase-12, and Bim in liver tissue of groups B and C mice were increased. Bcl-2 decreased (*P* < 0.05). Compared with group B and group C after naltrexone intervention, the expression levels of GRP78, IRE-1*α*, CHOP, caspase-12, and Bim decreased significantly, and Bcl-2 increased significantly (*P* < 0.05). Naltrexone can effectively reduce bodyweight and adipose tissue accumulation, reduce liver fat lesions, improve the expression of lipid metabolism-related proteins and endoplasmic reticulum stress, reduce liver lipid synthesis, and protect liver cells.

## 1. Introduction

The endoplasmic reticulum (ER) is an important organelle, involved in protein synthesis, secretion, and lipid synthesis. Endoplasmic reticulum stress (ERS) will be formed once exogenous substances, hypoxia, oxidative stress, and other reactions lead to ER imbalance. Cells activate signaling pathways through the unfolded protein response (UPR), including endoplasmic reticulum transmembrane protein kinase-1*α* (IRE-1*α*), to ensure ER stability [1–3]. Hepatic steatosis refers to the presence of fat droplets in the cytoplasm of liver stem cells. ERS will induce fatty degeneration, inflammation, or further apoptosis of liver cells. The clinical treatment of liver steatosis induced by endoplasmic reticulum stress mainly adopts lifestyle intervention and drug control, but there is no specific drug treatment [4, 5]. Naltrexone is an opioid receptor orange antiagent, which can improve the dependence of the body on alcohol, opioids, and so on by regulating the eating area of the brain and reducing the body mass. Based on this, we will analyze the effect of naltrexone on the expression of lipid metabolism-related proteins in liver steatosis induced by endoplasmic reticulum stress in mice. The experimental results are reported as follows.

## 2. Materials and Methods

### 2.1. Animal Data

30 inbred (C57BL/6J) male mice (Shanghai Slyke Company, China. Medical animal certificate number: 2015000518707) were placed in SPF grade animal room for one week after adaptive feeding, randomly divided into three groups, group A (control), group B (model), and group C (model + naltrexone), 10 animals in each group. The study was approved by the Affiliated Hospital of Zunyi Medical University.

### 2.2. Methods

#### 2.2.1. Major Reagents and Instruments

Naltrexone (Beijing Huasu Pharmaceutical Co., Ltd., China), 0.9% normal saline (Fuzhou Neptunus Pharmaceutical Co., Ltd., Shenzhen, China), high-fat feed (formulation: lard and sucrose 15%; cholesterol 2%; bile salt 0.5%; 67.5% feed) and conventional feed (Wu's Laboratory Animal Center, Fuzhou, China), ALT and TG test kits (Nanjing Jiancheng Technology Co., Ltd., Nanjing, China), BCA protein quantitative reagent box (Biyuntian, Shanghai, China), PCR kit (Applied Biosystems™ SYBR™ Green, Shanghai, China), xylene, absolute ethanol, and other solutions and primers (Biyuntian, Shanghai, China), related protein corresponding antibodies (CST, Danvers, USA), cryogenic centrifuge (Shanghai Luxiangyi Centrifuge Instrument Co., Ltd., Shanghai, China), nucleic acid protein analyzer (Beckman Coulter, Fullerton, CA, USA), and enzyme marker (Biyuntian, Shanghai, China).

#### 2.2.2. Group Modeling and Administration of Mice

After one week of adaptive feeding, 30 inbred (C57BL/6J) male mice were randomly divided into three groups, The mice were divided into group A (control), group B (model), and group C (model + naltrexone); each group of 10 mice were weighed and recorded once a week. The male mice in group A were fed with regular diet, and the mice in groups B and C were fed high-fat diet. Two mice were selected to establish a fatty liver model after 8 weeks of feeding. The histopathological sections were made to observe whether the model was established successfully. After the establishment of the model, a total of 24 mice, three groups of 8 mice, group A and group B were given an equal volume of 0.9% normal saline, intervention for 4 weeks, and the mice in group C were given naltrexone (100 mg/kg/d) by gavage at 8 o'clock daily according to their actual weight.

### 2.3. Specimen Processing

Liver tissue specimens were collected; 12 hours after the treatment, the mice were fasted and fixed under anesthesia. The weight, liver weight, and fat weight of mice were measured.

Blood samples were collected; 12 h after the course of fasting and under anesthesia, the mice were fixed. The abdominal cavity of mice was dissected. Cardiac blood was obtained by puncture of the diaphragm with a 5 ml syringe and placed in a 2 ml EP tube containing heparin. After 10 min, centrifugation (1500 r/min 20 min) was performed. The upper serum was placed in a low-temperature refrigerator to be tested.

### 2.4. Index Detection

Detection of mouse liver (alanine aminotransferase (ALT) and triglyceride (TC)) content, histopathological section (HE staining) to observe the grade of liver steatosis, and detection of lipid metabolism-related protein expression level: glucose regulatory protein (GRP78), endoplasmic reticulum transmembrane protein kinase-1*α* (IRE-1*α*), C/EBP homologous protein (CHOP), cysteine-containing aspartate proteolytic enzyme 12 (caspase-12), B lymphoma-2 (Bcl-2), and cell death mediator (Bim). All operations were performed in strict accordance with the standards in the kit.

### 2.5. Statistical Methods

Three groups of mice experimental data were included in SPSS 23.0 software analysis, and measurement data comparison is done using the *t*-test. The expression level of a protein associated with the lipid metabolism, ALT, and TG was expressed by (‾*x* ± *s*) (*P* < 0.05).

## 3. Results

### 3.1. Comparison of Bodyweight, Liver Weight, and Fat Weight in Three Groups of Mice

**Figure 1:**
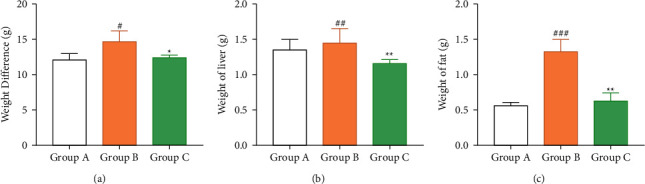
Comparison of bodyweight, liver weight, and fat weight among three groups of mice. ^#^*P* < 0.05, ^##^*P* < 0.01, ^###^*P* < 0.001, group A vs. group B; ^*∗*^*P* < 0.05, ^∗∗^*P* < 0.01, group B vs. group C.

Compared with group A, the bodyweight and fat weight of group B and group C were significantly increased (*P* < 0.05). Compared with group B, the bodyweight and fat weight of the mice were significantly decreased after naltrexone treatment (*P* < 0.05), as shown in Figure 1 and Tables 1 and 2.

### 3.2. The Levels of ALT and TG and the Degree of Steatosis in the Three Groups of Mice

**Figure 2:**
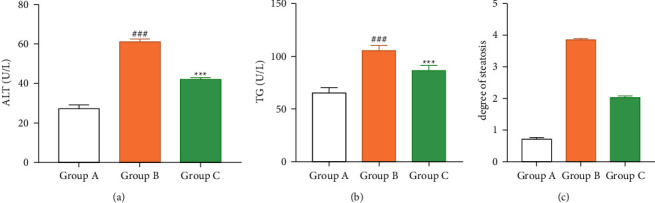
Comparison of ALT, TG levels, and degree of steatosis among three groups of mice.

Compared with group A, ALT, TG, and hepatic steatosis were observed in group B and group C (*P* < 0.05). Compared with group B, ALT and TG were significantly decreased and the degree of hepatic steatosis was reduced in group C, as given in Table 3 and Figure 2.

### 3.3. Expression Level of Lipid Metabolism-Related Protein in Three Groups of Mice

Compared with group A, the expression levels of GRP78, IRE-1*α*, CHOP, caspase-12, and Bim in liver tissue of groups B and C mice were increased. Bcl-2 decreased (*P* < 0.05). Compared with group B and group C after naltrexone intervention, the expression levels of GRP78, IRE-1*α*, CHOP, caspase-12, and Bim decreased significantly, and Bcl-2 increased significantly (*P* < 0.05), as given in Table 4.

## 4. Discussion

This study shows that eating high-fat diet can cause lipid metabolism disorder and related apoptosis, but naloxone can reverse the changes of the above indicators, suggesting that naloxone may be used as a clinical treatment for abnormal lipid metabolism diseases.

Severe hepatic steatosis may even lead to liver failure or stem cell necrosis, so clinical attention to the treatment of hepatic steatosis is high [6, 7]. Endoplasmic reticulum (ER) is one of the most important organelles in the body. Once exogenous substances, hypoxia, oxidative stress, and other reactions lead to the imbalance of ER, ERS will form [8–10]. Endoplasmic reticulum stress plays an important role in cardiovascular disease and hepatic steatosis. When this stress mechanism is disturbed by the intracellular environment, there may be a large accumulation of unfolded or misfolded proteins in the endothelium. The mechanism of inducing hepatic steatosis is as follows: GRP78 gene is closely related to the activation of the unfolded protein reaction pathway, so it is commonly used to detect the occurrence and extent of ERS. Activation of the IRE-1*α* gene can mediate activation of CHOP and caspase-12 and initiate ERS-mediated apoptosis. CHOP, JNK, and caspase-12 are the main apoptotic pathways mediated by endoplasmic reticulum stress. The apoptosis of hepatocytes in mice with hepatic fibrosis may be mediated by activating key signal molecules [11, 12].

The results of this study showed that compared with group A, after a high-fat diet, the bodyweight, fat weight, adipose index, ALT, and TG in groups B and C were significantly increased, and hepatic steatosis was observed (*P* < 0.05). Compared with group A, the expression levels of GRP78, IRE-1*α*, CHOP, caspase-12, and Bim in liver tissue of groups B and C mice were increased. Bcl-2 decreased (*P* < 0.05). The reason is that after a high-fat diet, mice will enter the liver with excessive lipid and fatty acid content, cause endoplasmic reticulum stress reaction, affect the related lipid metabolism-related protein levels, and continue to aggravate hepatic steatosis [13]. The pharmacodynamics of naltrexone is similar to naloxone. The mechanism of action is to weaken or block opioid receptors and then effectively relieve the body's dependence on opioids and keep a normal life. This product is effective for oral use, and the effect lasts a long time, and this product does not produce body or spirit dependence [14–16]. The mechanism of naltrexone treatment for steatosis is not clear, but the results of this study showed that compared with group B, after treatment with naltrexone in group C, the bodyweight, fat mass, fat index, ALT, and TG decreased significantly and the degree of hepatic steatosis decreased (*P* < 0.05). Compared with group B, group C after naltrexone intervention, the expression levels of GRP78, IRE-1*α*, CHOP, caspase-12, and Bim decreased significantly and Bcl-2 increased significantly (*P* < 0.05). It can be seen that naltrexone can improve endoplasmic reticulum stress response. Liver steatosis is a kind of reversible injury, and the original nature of liver fat can be restored after naltrexone eliminates the cause of the disease. The reason may be that naltrexone can reduce the stress on the endoplasmic reticulum and inhibit the degree of apoptosis of liver cells by interfering with the main apoptosis pathways of ERS mediated by IRE-1, CHOP, and caspase-12 pathways. In addition, naltrexone can also reduce the therapeutic effect of liver fatty disease by changing the phenotype and function of macrophages and other mechanisms.

In conclusion, naltrexone can effectively reduce bodyweight and adipose tissue accumulation, reduce liver fat lesions, improve the expression of lipid metabolism-related proteins and endoplasmic reticulum stress, reduce liver lipid synthesis, and protect liver cells.

## Figures and Tables

**Table 1:** Comparison of bodyweight of three groups of mice ($\bar{x}\pm s$, g).

Groups	*n*	Basic weight	Last weight	Weight difference (last weight–basic weight)
Group A	8	17.32 ± 1.45	28.58 ± 3.58	11.26 ± 2.13
Group B	8	17.29 ± 1.47	31.64 ± 3.75	14.35 ± 2.28
Group C	8	17.28 ± 1.46	29.65 ± 3.48	12.37 ± 2.02
*t*	—	—	—	4.260
*P*	—	—	—	0.028

**Table 2:** Comparison of liver weight and fat weight in three groups ($\bar{x}\pm s$, g).

Groups	*n*	Liver weight	Fat weight
Group A	8	1.41 ± 0.12^bc^	0.52 ± 0.14^b^
Group B	8	1.58 ± 0.17^ac^	1.26 ± 0.43^ac^
Group C	8	1.20 ± 0.13^ab^	0.57 ± 0.15^c^
*t*	—	14.450	18.080
*P*	—	0.001	0.001

Comparison between the two groups: Comparison with group A ^a^*P* < 0.05, ^b^Group comparison ^b^*P* < 0.05, with ^c^Group comparison ^c^*P* < 0.05.

**Table 3:** The levels of ALT and TG and the degree of steatosis in the three groups of mice ($\bar{x}\pm s$).

Groups	*n*	ALT	TG	Steatosis degree
Group A	8	27.26 ± 5.74^bc^	65.41 ± 11.32^bc^	0.72 ± 0.14^bc^
Group B	8	61.34 ± 13.28^ac^	105.58 ± 18.14^ac^	3.85 ± 0.74^ac^
Group C	8	42.35 ± 11.08^ab^	86.20 ± 7.19^ab^	2.03 ± 0.38^ab^
*t*	—	21.080	19.030	83.340
*P*	—	0.001	0.001	0.001

Comparison between the two groups: Comparison with group A ^a^*P* < 0.05, ^b^Group comparison ^b^*P* < 0.05, with ^c^Group comparison ^c^*P* < 0.05.

**Table 4:** Expression level of lipid metabolism-related protein in three groups of mice ($\bar{x}\pm s$).

Groups	*n*	GRP78	IRE-1*α*	CHOP	Caspase-12	Bim	Bcl-2
Group A	8	0.01 ± 0.01	0.02 ± 0.01	0.02 ± 0.01	0.01 ± 0.01	0.01 ± 0.01	0.06 ± 0.03
Group B	8	0.07 ± 0.02	0.08 ± 0.02	0.09 ± 0.02	0.06 ± 0.02	0.07 ± 0.02	0.02 ± 0.01
Group C	8	0.04 ± 0.01	0.03 ± 0.01	0.04 ± 0.01	0.03 ± 0.01	0.02 ± 0.01	0.05 ± 0.01
*t*	—	36.000	41.330	52.000	25.33 0	41.330	9.450
*P*	—	0.001	0.001	0.001	0.001	0.001	0.001

## Data Availability

The data used to support the findings of this study are available from the corresponding author upon request.
